# Questionnaire based evaluation of the motivation of surgically treated patients to participate in preventative hygiene measures

**DOI:** 10.3205/dgkh000336

**Published:** 2020-01-14

**Authors:** Daniel Philipp Exner, Meike Elisabeth Stoffels, Martin Exner, Steffen Engelhart, Jörg Christoph Kalff, Ricarda Maria Schmithausen

**Affiliations:** 1 Clinic for General, Visceral, Vascular and Thoracic Surgery, University Hospital Bonn, Bonn, Germany; 2 Institute for Hygiene and Public Health, University Hospital Bonn, Bonn, Germany

**Keywords:** nosocomial infection, knowledge, attitude, patients, involvement

## Abstract

**Background:** Nosocomial infections caused by antibiotic-resistant pathogens demonstrate the continued need for preventive hygiene management strategies. Information and training of patients in their personal hygiene is a current requirement of the German Society for Hospital Hygiene, and is recommended by the Commission for Hospital Hygiene and Infection Prevention (*Kommission für Krankenhaushygiene und Infektionsprävention beim Robert-Koch Institut*, KRINKO) at the Robert Koch Institute.

**Aim:** The aim of this study was to evaluate patients’ existing knowledge of hygiene and their motivation to actively participate in preventive hygiene measures.

**Methods:** This study included 445 inpatients at the Polyclinic for Surgery of University Hospital Bonn. Subjects were interviewed over a 6-month period using a questionnaire comprising 21 questions on the topic of hygiene.

**Results:** The majority of patients rated their subjective level of knowledge as intermediate (41%), 25% as poor and 35% as high. The respondents rated the active inclusion of patients in hygiene practices as highly relevant, and were willing to actively contribute to infection prevention, whereby the patients considered hand washing and hand disinfection in particular as important starting points. 78% of the respondents wanted more information on hygiene, particularly on wound and food hygiene. Targeted hygiene education provided by hospital staff had a positive effect on the patients’ subjective level of information, as well as on their confidence in physicians and nursing staff. Previous information via television or radio had a negative impact on the patients’ subjective information level and on their confidence in hospital staff.

**Conclusion:** Most surgically treated patients are motivated to actively contribute to preventive hygiene measures. This represents an additional and important option for applying hospital hygiene more effectively and, above all, closer to the patient. Information and education should preferably be performed by healthcare professionals.

## Zusammenfassung

**Hintergrund:** Präventive Strategien im Hygiene-Management stellen einen wichtigen Ansatzpunkt zur Vermeidung nosokomialer Infektionen durch Antibiotika-resistente Erreger dar. Die Information und Schulung der Patienten in ihrer persönlichen Hygiene stellt eine Forderung der Deutschen Gesellschaft für Krankenhaushygiene dar und wird ebenso von der Kommission für Krankenhaushygiene und Infektionsprävention beim Robert-Koch Institut (KRINKO) empfohlen.

**Ziel:** In der Studie sollte der Wissensstand von Patienten zum Thema Hygiene sowie deren Bereitschaft, aktiv an präventiven Hygiene-Maßnahmen teilzunehmen, evaluiert werden.

**Methode:** Über einen Zeitraum von 6 Monaten wurden 445 Patienten der Poliklinik für Allgemein-, Viszeral-, Thorax- und Gefäßchirurgie der Universitätsklinik Bonn mithilfe eines 21 Fragen beinhaltenden Fragebogens interviewt.

**Ergebnisse:** Die Mehrheit der Patienten schätzte den eigenen subjektiven Wissenstand zum Thema Hygiene als mittelmäßig (41%), 25% als schlecht und 35% als hoch ein. Die befragten Teilnehmer bewerteten die aktive Integration von Patienten in Hygiene-Maßnahmen als sehr wichtig und zeigten eine überdurchschnittlich hohe Motivation, an diesen Maßnahmen teilzunehmen. Dabei erschien den Befragten Händewaschen und Händedesinfektion am wichtigsten. 78% der Befragten wünschten sich mehr Information zum Thema Hygiene; von besonderer Bedeutung erschien den Teilnehmern das Thema Infektionsschutz bei der Wundversorgung und Lebensmittelhygiene. Wurden die Befragten bereits im Vorfeld durch medizinisches Fachpersonal über das Thema Hygiene informiert oder unterrichtet, zeigte sich ein positiver Einfluss sowohl auf den subjektiv eingeschätzten Kenntnisstand zum Thema Hygiene als auch auf das Vertrauen der Patienten in das ärztliche und pflegerische Personal. Erfolgte im Vorfeld die Information überwiegend über die Medien Fernsehen und Radio, zeigte sich ein negativer Einfluss sowohl auf den subjektiven Wissensstand als auch auf das Vertrauen gegenüber dem medizinischen Personal.

**Schlussfolgerung:** Der überwiegende Anteil der in der Chirurgie behandelten Patienten hatte ein überaus großes Interesse, an präventiven Hygienemaßnahmen teilzunehmen. Das stellt einen zusätzlichen wichtigen Ansatzpunkt für eine effektive, patientennahe und praktisch orientierte Krankenhaushygiene dar, wobei die Informationsvermittlung vorzugsweise durch medizinische Fachangestellte erfolgen sollte.

## Introduction

Nosocomial infections illustrate the continued need for improvement in preventive hygiene management. Integrating the patients themselves into actively participating in hygiene measures represents a widely neglected approach in the field of infection prevention. “Information and training of patients in their personal hygiene” is a current requirement of the German Society for Hospital Hygiene, and is also recommended by the KRINKO (Kommission für Krankenhaushygiene und Infektionsprävention beim Robert-Koch Institut) [1], [2]. Implementation of such measures requires information on patients’ knowledge and attitude towards hygiene. However, the data related to this is limited in Germany [2], [3], [4].

## Materials and methods

### Study design and questionnaire

From October 2014 to March 2015, 445 inpatients and their in-patient companions from the Department of General, Visceral, Thoracic and Vascular Surgery of the University Hospital Bonn took part in a questionnaire survey. All available inpatients during that period were included in the survey, except for those in isolation rooms, those who were not able to comprehend the questionnaire for linguistic reasons and patients not willing to complete the questionnaire. The participants were given a brief oral and written explanation of the reasons for the survey and the study protocol. Participants completed the paper-based questionnaire independently, then submitted the completed survey in prepared envelopes to collection points which had been set up especially for the survey.

The standardised questionnaire consisted of 21 closed questions. Questions were based on the following topics:

- relevance of hospital hygiene and patient integration;
- prior knowledge about hospital hygiene;
- level of confidence in physicians’ and nurses’ compliance with relevant hygiene measures;
- risk assessment for nosocomial infections;
- motivation to participate in the improvement of hygiene;
- further information request;
- personal information.

The answer options were scaled depending on the question: trichotomously grouped, multiple selection, or freely formulated. In addition, survey participants were given the opportunity to incorporate their own opinions and suggestions in a special field in the questionnaire for free formulation.

### Evaluation and statistical analysis

Statistical analysis of responses was performed using the Statistical Package for Social Sciences software (SPSS; version 22). The chi-squared and Mann-Whitney tests were used for data with two discretely distributed independent samples, and the Kruskal-Wallis test was used for data with more than two discretely distributed independent samples. The level of significance was set at an error probability of p≤.05. 

## Results

The response rate for the distributed questionnaires was 75% (332 out of 445). Personal information regarding the respondents is presented in Table 1 (Tab. 1).

Among the respondents who had been hospitalised within the past 10 years, 42% (n=318) reported having already received information about hygiene practices (especially hand hygiene) during their last stay in the hospital. The majority of patients rated their current level of knowledge as intermediate (41%), poor (22%) or very poor (3%). About a third rated their own knowledge on hospital hygiene as “high” (29%) or “very high” (6%).

A subjectively very high or high level of knowledge of hospital hygiene was highly significantly (p<0.001) associated with greater confidence in physicians and nursing staff (Table 2 (Tab. 2)).

Patients’ confidence in hospital staff also had a highly significant influence (p<0.001) on their fear of suffering from nosocomial infections (Table 3 (Tab. 3)). The more confidence patients had in the staff, the less anxiety they had about being affected by nosocomial infections. Confidence in physicians and nursing staff, in turn, was significantly influenced by the type of information media patients used in advance. Table 4 (Tab. 4) shows the influence on the subjective level of knowledge, confidence in physicians and nurses, and fear of nosocomial infections according to the previously used information source. While there was no significant influence of a previous hospital treatment within the last 10 years or a nosocomial infection that the patients themselves had suffered from, knowing a person who suffered from a nosocomial infection or age >60 years increased the fear that the patients could be affected by themselves. 

If flyers and brochures were used in advance as an information tool on the topic of hygiene, this type of media had a significantly positive impact on the subjective level of knowledge of hospital hygiene topics, in contrast to newspapers, television and radio. Indeed, the latter information sources seemed to have a significantly negative impact on the subjective level of knowledge, confidence in physicians and nurses, and fear of nosocomial infections among respondents (Table 4 (Tab. 4)). Previously informating patients by hospital staff had a particularly positive impact on the subjective level of knowledge (+19% “very high”, +26% “high”; p<0.001) and confidence (+19% “very high”; p=0.007; Table 4 (Tab. 4)). Internet use had no significant influence on the subjective level of knowledge, confidence and fear of the patients.

As far as the patients had already been informed in advance about the topic by their general practitioner, patients were significantly more willing to implement wound (+26%, p=0.013) and catheter hygiene (+29%, p=0.004). 

Of the respondents, 73% considered that inclusion of patients in hygiene measures was “very important” and 26% considered it as “important”, while the patients’ level of school education did not have a relevant influence. Furthermore, 37% of the respondents were “very much” and 40% “somewhat” of the opinion that more information about self-performed hygiene measures could positively affect their recovery. Almost all participants (98%) stated that they were willing to actively contribute to infection prevention through their own personal hygiene behaviours. This was particularly applicable to hand disinfection (90%), more frequent hand washing (83%), habitual changes in the sanitary area (62%), avoiding handshaking (59%), and wound care (53%). 

Of the respondents, 78% desired more information about hygiene, especially about wound care (61%), food hygiene (59%) and hygiene in the sanitary area (58%), but less about hand (46%) and catheter hygiene (38%). 

The participants preferred oral information provided by hospital staff (69%), written information in brochures/flyers (59%) and videos shown in the waiting area (44%) or in the patient room (41%) (Table 5 (Tab. 5)). 

## Discussion

So far, the available data on patients’ subjective level of knowledge on the subject of hygiene is limited, while in this survey two-thirds of the respondents described their subjectively assessed level of knowledge as moderate or even good.

In addition, the patients interviewed were very motivated to receive information about how to actively contribute to infection prevention, and expressed a desire to be involved in hygiene measures. A good subjective knowledge was found to be correlated with increased confidence in physicians and nursing staff.

The willingness of patients to contribute to infection prevention through their personal hygiene behaviour was generally very high. However, the actual practical implementation of these measures could not be verified or tested in the current study. The interest in hygiene was usually focussed on the patient’s own suffering. For example, the surgically treated patients surveyed here expressed a strong desire for information on hygiene related to wound care. Thus, more specialised hygiene practices, like proper utilisation of urinary or central venous catheters was not relevant to a relatively large proportion of the patients.

46% of the respondents stated that information about hand washing and disinfection was useful. This is supported by a representative study on hand hygiene which found that the recommended duration of hand washing (20 seconds) was only achieved by 36% of respondents [5]. Therefore, demonstration of correct hand disinfection procedures based on (digital) images and targeted to patients would be useful. Gagné et al. [6] demonstrated that active motivation through a hand disinfection demonstration reduced the methicillin-resistant *Staphylococcus aureus *(MRSA) infection rate by 51%. In particular, the incidence of septicaemia and respiratory tract infections decreased. Barker et al. [7] and Pittet et al. [8] showed that 89% and 85% of patients, respectively, were more likely to use hand disinfectants if they were located close to the bed.

The way in which information is conveyed is crucial for obtaining a subjectively good level of knowledge. For example, oral information provided by hospital staff (physicians and nursing staff) and written information in flyers was better accepted than through other media (Table 5). Hospital staff can respond directly to patients, answering questions and addressing their fear of nosocomial infections based on the individual’s prior knowledge. 

A disadvantage of this source of information is that the additional working time spent on individual information measures has so far hardly been reflected in the regular working time models, which is a distinct barrier to involving patients and their relatives in hospital hygiene [9]. On the other hand, integrating the patient in proper use of hygiene measures could offer a time-saving option, if undertaken in the right way. 

According to Jung [10], the rate of understanding and subsequent implementation of information when solely reading (10%) or listening (20%) is very low. This can, however, be increased to 50% by seeing and hearing the information, or to 90% by self-application. Therefore, it makes sense not only to provide oral and written information, but also to provide video sources designed in collaboration with medical professionals. Such a combination is also supported by the fact that patients rated an average of 2.8 different forms of information as optimal. This means that actions such as hand washing and hand disinfection should be performed correctly at least once under guidance, and could then be performed repeatedly by the patients while following video instructions.

The informative value of the present patient survey is limited by the fact that only surgical patients and their in-patient companions were allowed to take part in the study. On the one hand, the results could be better interpreted due to the relative homogeneity of the circumstances among the respondents. On the other hand, it remains to be clarified how patients of other clinical disciplines would answer as the topic of hygiene is increasingly accepted by the society. 

## Conclusions

In principle, the motivation of surgically treated patients to contribute to infection prevention through their personal hygiene behaviour was very high. Furthermore, participants reported a desire for additional information about hospital hygiene. Of central importance is that education of patients already provided by hospital staff was found to be associated with a subjectively better level of knowledge, as well as with greater confidence in physicians and nursing staff regarding compliance with the required hygiene measures. Hand hygiene remains an important starting point as patients already felt well informed about it and were motivated to improve their hand hygiene skills.

## Notes

### Competing interests

The authors declare that they have no competing interests.

### Acknowledgements

We sincerely thank the patients of the University Hospital Bonn who took part in the survey. We also thank the nursing staff for supporting the study.

## Figures and Tables

**Table 1:**
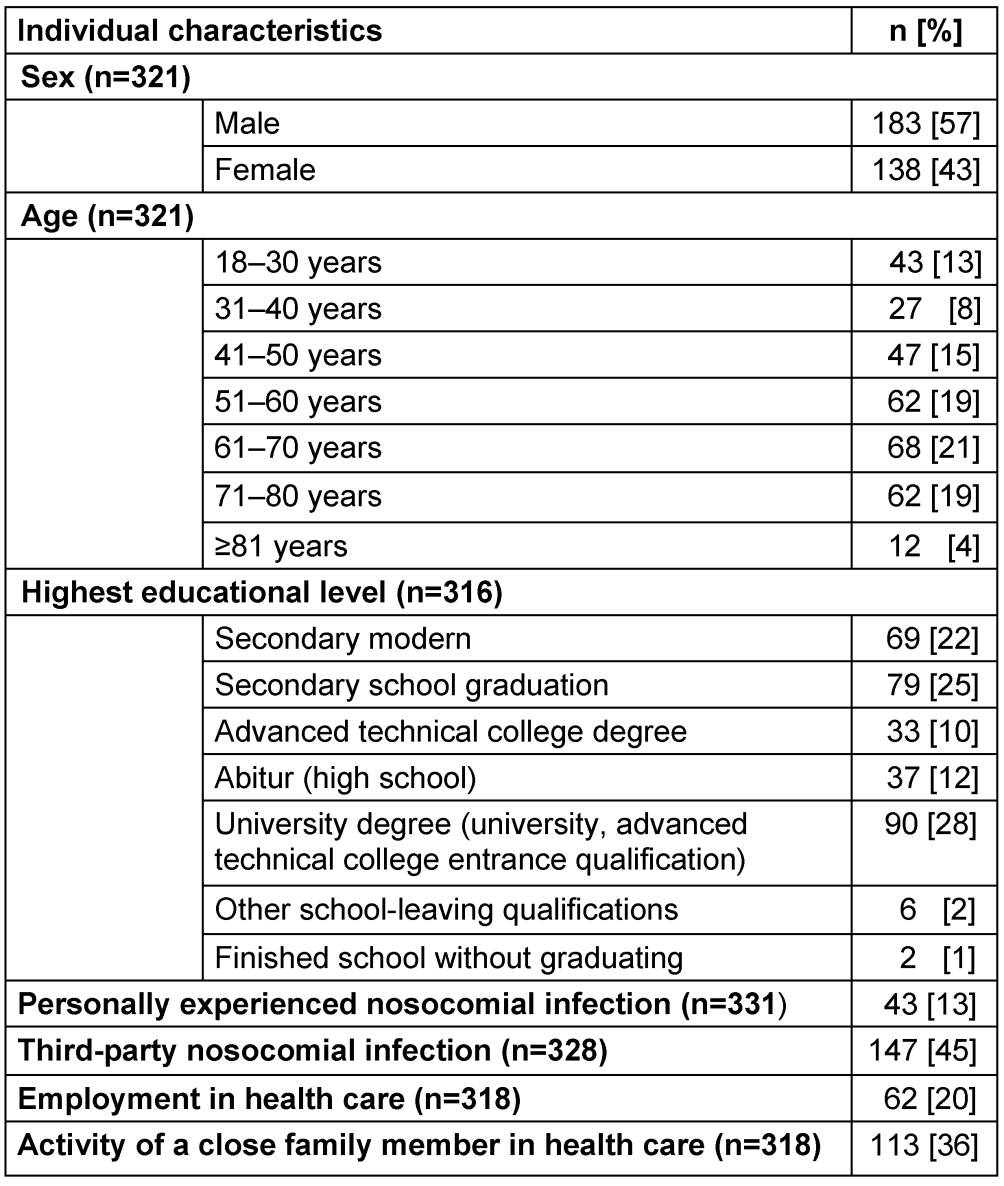
Distribution of individual characteristics of the responding patients (n=332)

**Table 2:**
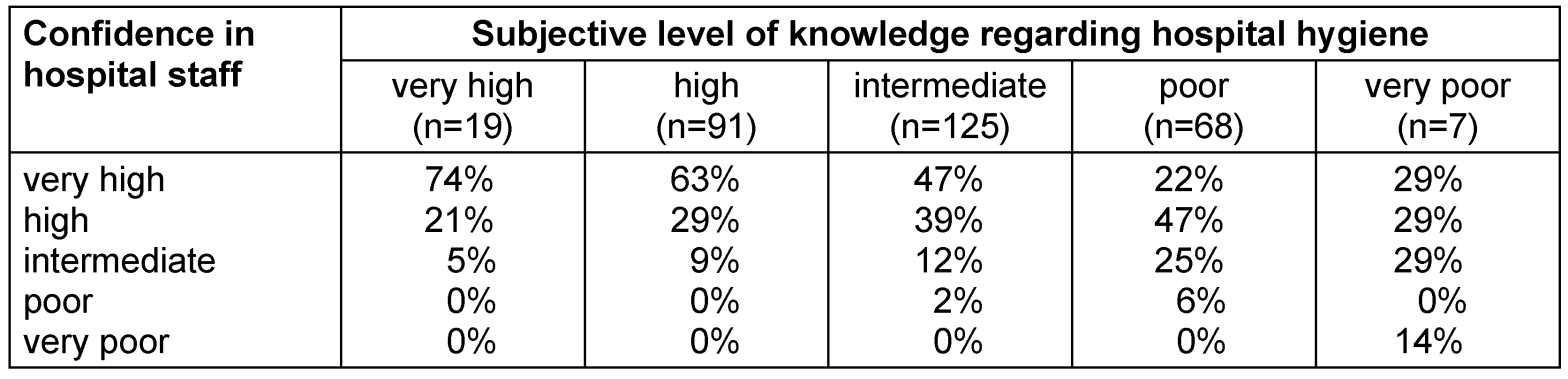
Confidence of participants in attending physicians and nursing staff to comply with the required hygiene measures depending on their subjective level of knowledge regarding hospital hygiene (n=310)

**Table 3:**
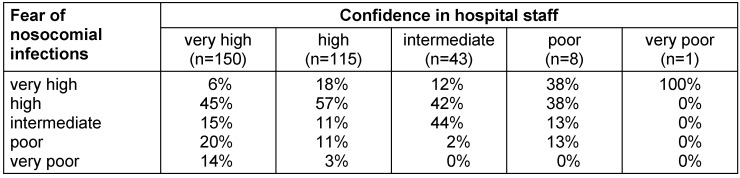
Fear of nosocomial infections depending on the confidence of participants in attending physicians and nursing staff to comply with the required hygiene measures (n=317)

**Table 4:**
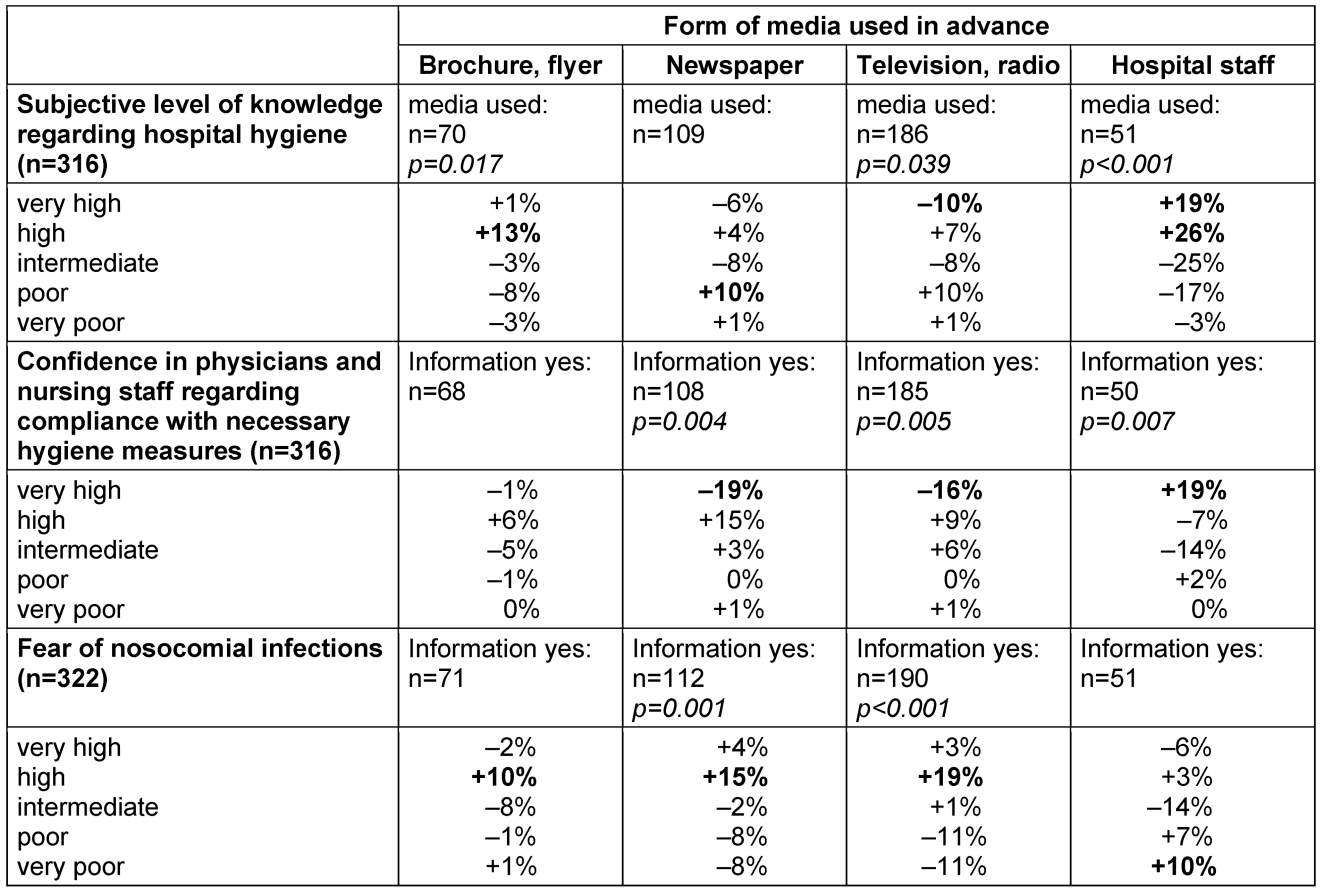
Influence of sources of information used on the confidence of participants in physicians and nursing staff regarding compliance with necessary hygiene measures (n=316), fear of nosocomial infections (n=322), and their subjective level of knowledge of hospital hygiene (n=316). Several sources of information could be selected. The percentages indicate the difference between participants who did and did not use the respective information source for each characteristic (difference “yes” vs. “no”). Significant results are indicated.

**Table 5:**
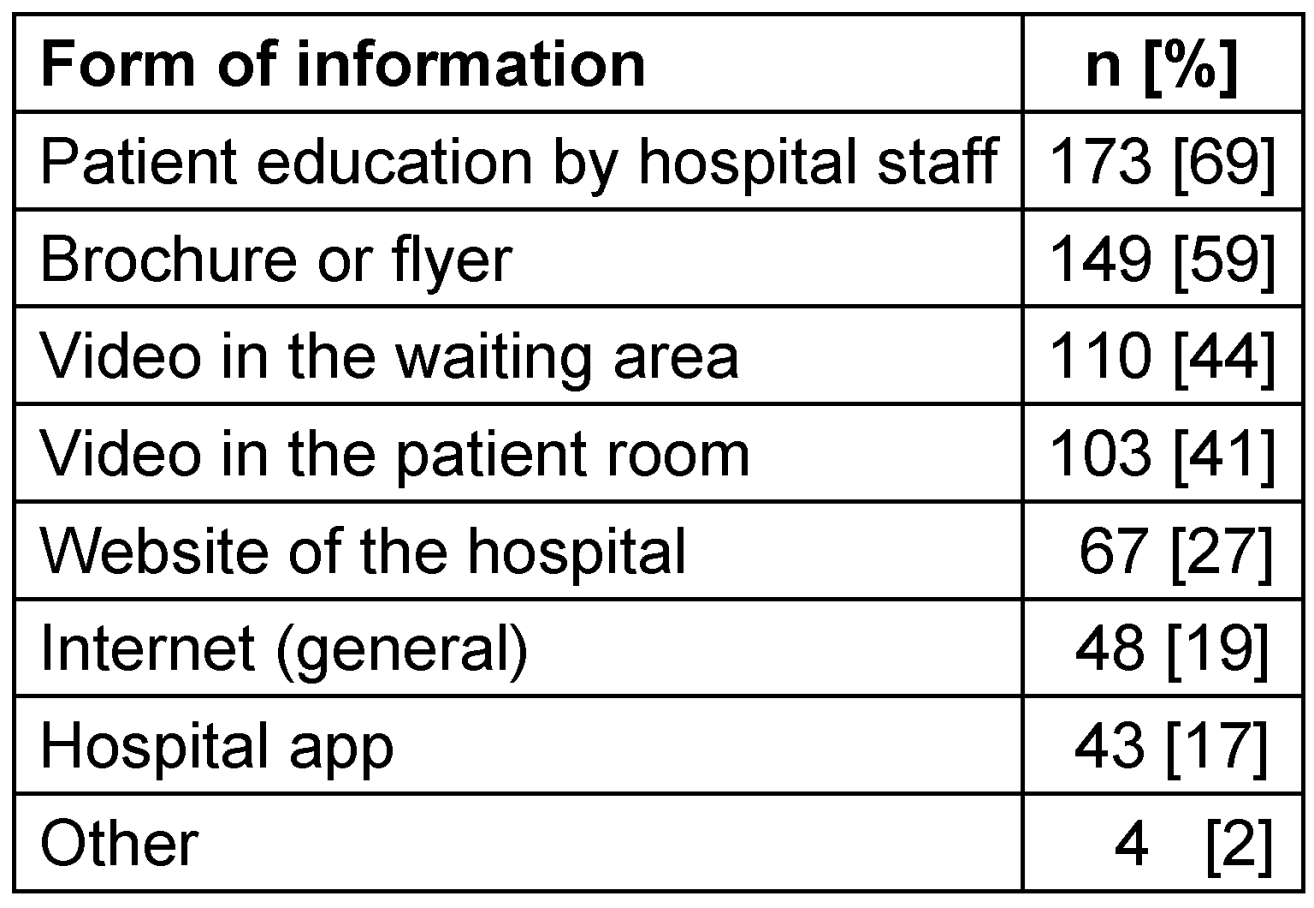
Preferred form of information on hospital hygiene from patients’ subjective perspective (n=251)
